# Early Detection of Parkinson’s Disease Using AI Techniques and Image Analysis

**DOI:** 10.3390/diagnostics14232615

**Published:** 2024-11-21

**Authors:** Marilena Ianculescu, Corina Petean, Virginia Sandulescu, Adriana Alexandru, Ana-Mihaela Vasilevschi

**Affiliations:** 1 National Institute for Research and Development in Informatics, 011455 Bucharest, Romania; marilena.ianculescu@ici.ro (M.I.); corina.petean@ici.ro (C.P.); ana.vasilevschi@ici.ro (A.-M.V.); 2 Faculty of Electrical Engineering, Electronics and Information Technology, Valahia University of Targoviste, 130004 Targoviste, Romania

**Keywords:** Parkinson’s disease, image analysis, Parkinson’s disease detection, Archimedean spiral

## Abstract

Background: Parkinson’s disease (PD) diagnosis benefits significantly from advancements in artificial intelligence (AI) and image processing techniques. This paper explores various approaches for processing hand-drawn Archimedean spirals in order to detect signs of PD. Methods: The best approach is selected to be integrated in a neurodegenerative disease management platform called NeuroPredict. The most innovative aspects of the presented approaches are related to the employed feature extraction techniques that convert hand-drawn spirals into a frequency spectra, so that frequency features may be extracted and utilized as inputs for various classification algorithms. A second category of extracted features contains information related to the thickness and pressure of drawings. Results: The selected approach achieves an overall accuracy of 95.24% and allows acquiring new test data using only a pencil and paper, without requiring a specialized device like a graphic tablet or a digital pen. Conclusions: This study underscores the clinical relevance of AI in enhancing diagnostic precision for neurodegenerative diseases.

## 1. Introduction

Parkinson’s disease (PD) is the second most common neurodegenerative disorder (after Alzheimer’s Disease) [1] and it is the fastest-growing neurological disorder in terms of disability [2]. PD presents a wide range of symptoms, including motor symptoms (such as bradykinesia, tremor, and rigidity) and non-motor symptoms (such as cognitive impairments, sleep disturbances, and pain) [1,2], which progressively worsen over time. Although there is no cure for PD, managing its symptoms is of the essence for maintaining a good quality of life and independent living. For this reason, early diagnosis is crucial.

Diagnosing PD is a challenging task, as there is no single definitive standardized test that clearly detects the presence or absence of the disease. Diagnosis is primarily based on the clinical evaluation performed by a neurologist. The assessment includes an analysis of medical history, observation of motor symptoms, and response to specific medication. Imaging and other tests may be used to rule out other conditions. Even though scales for rating the severity of PD exist, the most widely used are the Unified Parkinson’s Disease Rating Scale (UDPRS) and its variants (like MDS UDPRS) [3,4], these must also be applied and assessed under the supervision of specialized personnel. For example, the study presented in [3] shows that there are statistically significant differences in the results of these standardized tests when performed by nurses or by neurologists, depending on the level of expertise of the performer.

PD, even in early stages, is associated with movement disorders like tremor, rigidity, bradykinesia. In order for a person to be diagnosed with PD, bradykinesia and either rigidity or tremor must be present. These symptoms reflect in the way a person is able to write and draw.

A simple test for PD detection is based on the analysis of hand-drawn Archimedean spirals. The authors of [5] demonstrated that spiral analysis correlates with the motor part of the UDPRS and may be useful in the assessment of Parkinsonian signs. The test is usually performed in a clinical setting, where the drawing is assessed by specialized personnel with the expertise to put the results in perspective and correlate them with other relevant data from medical history and other different tests. There are a multitude of studies that try to automatically detect signs of PD in hand-drawn spirals [5,6,7,8].

Early studies focused on visually analyzing the drawn spirals and the behavior of the subjects while drawing, subsequent studies focused on using digital tools to more precisely analyze spiral drawings, and more recent studies have introduced machine learning (ML), including deep learning (DL) techniques for automatically detecting signs of the disease.

Ongoing studies continue to refine the techniques for analyzing spiral drawings and explore their correlation with other clinical measures. The goal is to enhance early detection and monitoring of PD, ultimately improving quality of life and health outcomes for PD patients.

The main aim of this study is to develop a binary classification algorithm that reliably detects signs of PD based on hand-drawn spirals. This algorithm is to be integrated in a complex application for managing neurodegenerative diseases, including PD. This paper presents the steps employed in building the algorithm. It is designed to work only on the spiral images, without requiring additional information like drawing speed, in-air and on-surface timings, number of pauses, and other information that may only be available if the drawing is produced using specialized devices that are not widely available, like digital tablets or pens. This way, tests on new images may be performed using a regular pencil and paper, without needing a graphic tablet or digital pen. This requirement comes from the need to integrate the algorithm in a PD screening tool that must be available to use through the NeuroPredict [9] platform in different testing settings, not just a clinical setting.

NeuroPredict was designed and is currently being developed as one of the results of the project “Advanced Artificial Intelligence Techniques in Science and Applied Domains”, a project in its second year of implementation out of a total length of four years. The NeuroPredict platform integrates multiple predictive modeling algorithms customized for three specific disease, PD, Alzheimer’s Disease, and Multiple Sclerosis, that rely on data provided by Internet of Medical Things devices integrated in the subject’s living environment or worn by the monitored subjects. The platform integrates AI algorithms based on the acquired physiological information and self-reported data and is designed to deliver accurate and timely insights that have the potential to impact neurodegenerative disease management and comprehension. The PD detection algorithm presented in the current work is one of the many tools designed to be integrated in the NeuroPredict platform that may be used for screening and monitoring PD subjects or patients at risk.

The most innovative aspect of the presented work lies in the feature extraction phase, where the spiral images are converted into signals that may be further processed in both the time and frequency domains. The algorithm also uses features related to the weight of the line and its variations. The extracted features are grouped in two main categories called frequency features and pencil features. The features are then used to build multiple ML-based classifiers and the model with best performances in terms of accuracy, precision, and recall is selected to be integrated in NeuroPredict, the platform for managing neurodegenerative diseases.

As previously mentioned, the current work explores multiple approaches for developing the PD classification algorithm, with various feature sets and employing multiple ML techniques, described in Section 2, Materials and Methods. For comparison with other works selected from a literature review, it is important to mention that in the current work, the best results were achieved using Random Forests (RFs). The average accuracy for the best model is 94.59%, as will be further detailed in the Results Section.

Table 1 presents a summarization of other works in the area of PD detection from hand-drawn spirals, selected by novelty criteria, dataset used, and data acquisition modality.

As seen in Table 1, there is a dominant trend in recent studies for using DL methods, particularly variations of DenseNet, showcasing high accuracy levels. In [8], the authors use DenseNet and ResNet variations to discriminate between healthy and PD patients using the dataset presented in [7] augmented several times, achieving the highest accuracy found in the literature review using DL, 96%. This accuracy is reported for the algorithm trained for 50 epochs and it is stated that the same algorithm, when trained for 100 epochs, achieves a lower average accuracy of only 89%. This might suggest that the high accuracy achieved at 50 epochs was measured before the learning curve stabilized and that the classifier built for 50 epochs might not perform well on new data, or it may suggest that if trained for 100 epochs, overfitting occurs. Learning and loss curves are not provided to clarify this aspect. Although DenseNet achieves good results, 96% in [8] and 94% in [10], there are disadvantages presented by DL techniques, compared with the classification technique selected for current work (RFs). These drawbacks stem from the fact that they are highly complex models that require significant computing resources for training the models and also for inference on new data.

The main purpose of the work presented in this paper is to develop an algorithm that reliably detects PD from hand-drawn spirals that may be integrated into a complex application for remote monitoring and management of neurodegenerative disease patients and people at risk, including PD patients. For the algorithm to be appropriate for the required usage scenario, it must meet two criteria: (a) demonstrate good discrimination performance and (b) rely on data that may be acquired without specialized equipment. The motivation for (b) is that the proposed PD testing is intended to be used in different environments, not only in clinical settings. Even in clinical settings, it is preferable to avoid the need for dedicated equipment when testing new subjects. To achieve this, the presented algorithm is designed to work directly on images of hand-drawn spirals, without requiring additional data like timing information for each point of the drawing or in-air timings. For the same reason, each work from the literature review has been analyzed to check if it meets this requirement, as indicated in the last column of Table 1. Based on the input data, two major categories are identified: (a) classifiers that work on data collected using only traditional methods (pen and paper) and (b) classifiers that require data recorded using devices that acquire in-air or/and on-surface kinematic variables, which may only be obtained using digital devices like graphic tablets, digital pens, and other devices with tactic screens. The best reported accuracy in the literature review is presented in [14], where multiple classification techniques are used on data recorded with a digital tablet. The extracted features comprise drawing speed, acceleration, and in-air and on-surface time, among others, which are available only when a graphic tablet or a special pencil is used for recording the input data.

The structure of the paper is as follows: Section 2 provides a clear presentation of the steps employed in building the algorithm. It provides a description of the dataset used, the augmentation techniques employed to enrich the dataset, the steps for extracting the frequency features, and the pencil-related features. Afterwards, it presents descriptions of the models built in order to find the most appropriate technique for the dataset and the extracted features by presenting the classification models and the used evaluation metrics, using different approaches: multiple ML techniques and different subsets of the extracted features. Section 3 presents the results obtained with each ML technique on each of the feature subsets. Section 4, Discussions, presents the limitations of the approach and interpretations of the results and puts them in perspective comparing them to other works in the field. The final section, Conclusions, presents the implications of the presented work and future work directions.

## 2. Materials and Methods

This study presents a novel approach for detecting PD using spiral drawings, which differs from existing methods that rely on digital devices to measure additional real-time characteristics such as speed and pressure. The proposed method exclusively uses traditional tools—paper and pencil—to collect data, focusing on extracting meaningful information from the drawing converted into a signal in the frequency domain and the inherent variations in pencil content, such as pencil thickness and pressure, as part of the drawing’s physical attributes. The frequency domain conversion allows analyzing both the signal and noise content, reflecting the specific characteristics of the tremor associated with PD.

The smoothness and consistency of the spiral are reflected in the frequency analysis without needing direct measurement. The proposed image-to-frequency conversion method simplifies the process by analyzing digitized versions of paper-and-pencil drawings, allowing for efficient detection without the need for additional real-time measurements.

**Figure 1:**
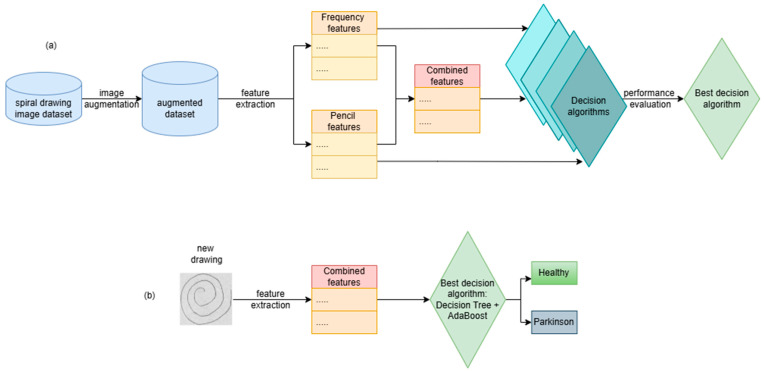
Pipeline for (**a**) building the decision algorithm and (**b**) using the decision algorithm.

The steps for building the PD detection algorithm based on hand drawings of Archimedean spirals are presented in Figure 1a. Figure 1b presents the steps for utilizing the decision algorithm in order to make predictions/decisions on new images. New images are processed in order to compute the relevant features that are fed to the decision algorithm in order to make a decision: the subject that drew the image is healthy or suffers from PD.

The steps depicted in Figure 1a are presented in detail in the following sections.

### 2.1. Description of the Dataset

The literature review presents only a limited number of datasets containing images of hand-drawn spirals produced by both healthy subjects and patients with PD. Access to the PaHaW [17] dataset, possibly the most used dataset found in the literature review, is conditioned by a license agreement that strictly limits its usage for research purposes. The purpose of this work goes beyond validating the viability of detecting PD from hand-drawn spirals to creating a model that may be used in real-world scenarios to aid in PD screening and diagnosis, so the usage of PaHaW was dismissed. Future work may look at using the dataset for validation of the classification method purposes only. Another dataset frequently used in this area is the University of California, Irvine Parkinson’s disease spiral drawings [11], which may be also used in future work for both improvement and validation of the built classification model.

The dataset used in the current work is presented in [7]. It contains drawings of Archimedean spirals produced by 55 subjects: 28 from a control group, healthy subjects, and 27 PD patients. The dataset contains 102 spirals: 51 spirals drawn by subjects from the control group and 51 spirals drawn by PWP subjects. The control group was chosen to be demographically similar with the PWP group, considering gender and age, in order to minimize differences between the groups that may be related to other aspects than PD. For the PD patients who were on levodopa treatment, the experiments were conducted while they were in the “on” stage. As examined using the UDPRS, on the Hoehn and Yahr Modified Scale [18], none of the PD patients were assessed to be in a late stage of the disease.

The drawings were captured using a digital drawing tablet with a dedicated pen in order to obtain the image itself and the drawing speed and pressure as time series with a recording frequency of 133 Hz. Participants were asked to draw guided Archimedean spirals and waves. In the current study, only the images containing the spirals are used. The guiding spiral consisted of 4.5 revolutions, had a maximum radius of 75 mm, was sketched in the clockwise direction, and was shown to the participants as dots spaced 2 mms apart. The final images contain only the drawings made by the subjects and no dots from the guiding spiral.

### 2.2. Dataset Augmentation

Different augmentation techniques were considered in order to enrich the dataset. The usual techniques for augmenting image datasets comprise the following: rotation, shifting, blurring, brightening, and scaling with subunitary and supraunitary factors, among others. The only augmentation technique that was deemed appropriate was rotation. Scaling was dismissed, as the dimension of the drawn spiral might contain information related to the targeted condition, as several studies concluded that PD patients tend to draw smaller and more compact spirals (micrographia) [1,8]. Shifting was dismissed because both feature extraction pipelines (for pencil-related features and for frequency features) rely on detecting the center of the spiral, and its position on the drawing relative to the margins of the drawing carries no significance, blurring was dismissed considering that it may hide meaningful information from images, and brightening was dismissed as both pipelines for feature extraction work on the masked image, were original luminosity is removed.

**Figure 2:**
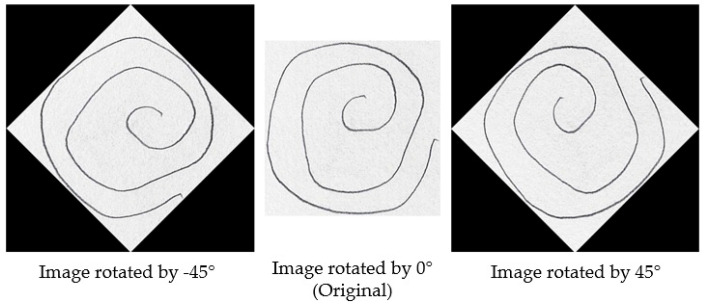
Examples of augmented spiral images rotated at various angles.

To illustrate the augmentation process, Figure 2 presents a set of images generated through rotation at various angles. As described earlier, each original image was augmented by applying rotations between −45° and 165° at 15-degree intervals. Figure 2 showcases examples of augmented images at selected angles, including −45°, 0°, and 45°. It is important to note that the image at 0° represents the original, unaltered image, while the other images demonstrate the effects of rotation at various angles.

Following augmentation, the process continued with the extraction of frequency domain features, pen-related features, and combined features, which were computed and stored in separate comma-separated values (csv) files. Each csv file contained the label for each image and the corresponding feature values.

For each spiral, two main types of features were extracted:

1. Frequency-based features: These features are derived by converting the spiral drawings into frequency spectra using Fast Fourier Transform (FFT). This allows the quantification of the frequency and amplitude of tremors, which are characteristic of PD. Several metrics from the frequency domain are computed, including the peak frequency, peak magnitude, and signal-to-noise ratio (SNR).
2. Pencil-based features: These features are extracted from the grayscale intensity of the spirals. Mean pixel intensity and variance in line thickness are computed.

### 2.3. Feature Extraction: Frequency Features

#### 2.3.1. Image-to-Frequency Conversion Method

**Figure 3:**
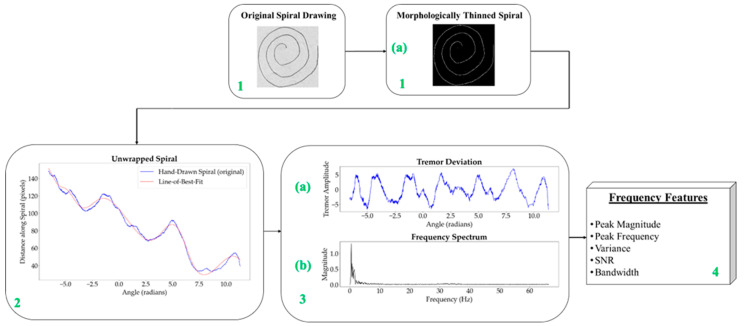
Image-to-frequency conversion process for spiral drawings.

The process for obtaining the frequency information is methodically structured, as illustrated in Figure 3, and each step is clearly outlined in Table 2 to ensure replicability and accuracy in identifying Parkinsonian tremors.

To effectively analyze the spiral drawings, the mathematical model of the Archimedean Spiral is used, which is defined in polar coordinates (*r*, *θ*) as follows:

(1) r=aθ,

where *r* is the length of the radius from the origin, *a* is a constant, and *θ* controls the number of rotations in radians. The radius length is a fundamental component of polar coordinates, indicating the distance from a specific point on the spiral to the origin. As the angle *θ* increases, the radius *r* also increases, causing the point to move away from the center in a spiral pattern. The constant *a* determines the rate at which the spiral expands away from the origin as it rotates around the center. This uniform and proportional increase in the radius with the angle makes the Archimedean spiral particularly suitable for scenarios where such growth needs to be measured. In the context of the present study, this model provides a solid foundation for quantifying tremor deviations, which are indicative of PD. The mathematical model of the Archimedean spiral [19] serves as the basis for the unwrapping process described in the next section.

Starting with a raw image, as shown in Figure 3 (block 1), the first step involves converting the spiral drawings into single-pixel-width sketches. This morphological thinning simplifies the spirals, making it easier to perform a pixel-by-pixel analysis along the spiral path in order to unravel the drawing into a linear representation. A one-pixel-width sketch facilitates precise measurement of tremor deviations along the spiral, which is essential for accurately identifying Parkinsonian tremors.

#### 2.3.2. Morphological Thinning

**Figure 4:**
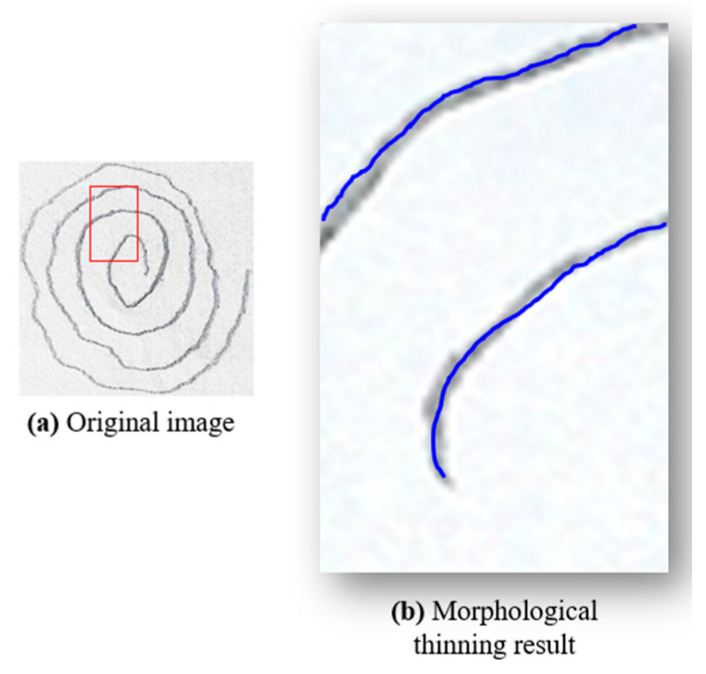
The process of morphological thinning and edge detection results on a spiral drawing showing (**a**) the original image from the dataset with the red rectangle marking the region of interest that is presented in (**b**) the processed image with the selected morphological thinning algorithm.

To achieve an accurate one-pixel-width contour of the spiral drawings, various image processing techniques can be employed, including skeletonization, medial axis skeletonization, and morphological thinning. In this study, we utilized morphological thinning because it produces a one-pixel-width image with minimal unnecessary branches, which is essential for accurate tremor analysis in PD detection. This method was chosen over other techniques, such as curvature detection or edge detection algorithms, which often produce artifacts like closed loops or double contours—two parallel lines, one on the outer and one on the inner boundary of the shape. These artifacts occur because these algorithms interpret the edges by creating lines on both sides of the actual boundary, leading to complex patterns that complicate the analysis. In contrast, morphological thinning removes redundant pixels that do not contribute to the essential trajectory of the spiral, reducing noise and potential distortions. This step is crucial for preparing the image for further processing, such as unwrapping and detailed frequency analysis, by retaining only the core path of the spiral. Morphological thinning was implemented using the OpenCV library [20], applying the Zhang–Suen algorithm [21] to transform thick lines into thin, one-pixel-width lines. As shown in Figure 4, morphological thinning (Figure 4b) provides a clear and accurate representation of the spiral’s core path.

#### 2.3.3. Spiral Unwrapping

**Figure 5:**
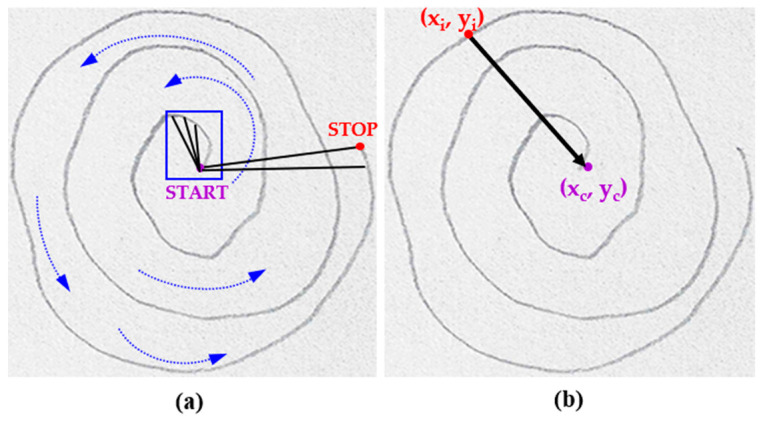
Unwrapping process of the spiral drawing by calculating the distance from the center at each pixel location: (**a**) depicts the START point in purple and the STOP point in red (**b**) shows the distance from an arbitrary point on the spiral (red) to the center of the spiral (purple).

In the process of drawing a spiral, the starting point is defined when the pencil first touches the paper, and the endpoint is reached when the pencil lifts, completing the spiral’s path. For studies utilizing graphic tablets to collect spiral drawings, a time component is measured as the drawing progresses, capturing the motion from start to finish, and data are captured as time series. In contrast, for paper-and-pencil sketches, the drawings must be interpreted considering the positions of pixels without any time-related information. To achieve this, the thinned image is used to define the start point (*x_c_*, *y_c_*) as the central pixel of the spiral. The unwrapping process involves counting each pixel (*x_i_*, *y_i_*) from this start point to the endpoint, calculating the Euclidean distance to the center at each step. The Euclidean distance, defined as

(2) $$d=\sqrt{{\left(x_{i}-x_{c}\right)}^{2}+{\left(y_{i}-y_{c}\right)}^{2}},$$

is computed for each pixel along the spiral path, where 0 ≤ *i* ≤ total length of the spiral. This iterative counting and distance calculation effectively and accurately “unwraps” the spiral into a linear form, as illustrated in Figure 5. The central pixel, marked as the START point in purple, and the endpoint, marked as the STOP point in red (Figure 5a), represent the boundaries of this unwrapping process.

To implement this unwrapping step, the unprocessed image is first converted to grayscale to simplify the analysis, as color information is not relevant in this context. A Gaussian filter is then applied to reduce noise and smooth out fine details that could introduce errors in subsequent processing steps.

Following filtering, adaptive thresholding is used to differentiate the spiral from the background, converting the image into a high-contrast black-and-white representation where the spiral becomes the focal point of analysis. The largest contour representing the spiral’s path is extracted. The central pixel is calculated based on this contour as the starting point of the drawing, to serve as a reference point for further analysis. The coordinates of the contour are transformed from Cartesian to polar coordinates, allowing for a clear representation of the spiral’s unwound path in terms of radius and angle. This transformation is essential for maintaining the correct directional angles around the center and ensuring a smooth transition in the graphical representation. Finally, the sorted distances and angles form the basis for the angle–distance plot, which visualizes and facilitates further analysis of the unwrapped spiral.

#### 2.3.4. Tremor Deviation

In the analysis of hand tremor, particularly for assessing PD, tremor deviation refers to the measurement of irregular movements that occur when an individual attempts to draw a spiral. To quantify this deviation, an “ideal” reference model of the spiral is required. This reference is obtained by unwrapping the hand-drawn spiral and comparing it to a fitted curve, often referred to as the LOBF. Spline fitting was employed to create a smooth curve that represents the ideal spiral path. This method was chosen for its flexibility in modeling rounded contours while minimizing the introduction of artifacts, such as excessive oscillations, that could obscure true tremor deviations.

The deviation is then calculated as the difference between the actual unwrapped spiral path and the idealized curve. This approach allows for precise quantifications of deviations, which directly reflect the irregularities in hand movements associated with Parkinsonian tremors.

The tremor deviation analysis utilizes the previously calculated radial distances and polar angles from each contour point of the spiral to the centroid. These measurements serve as the basis for constructing the coordinate pairs needed to analyze tremor characteristics. To smooth the data, cubic spline fitting is applied, with a smoothing parameter *s* that is carefully adjusted. The cubic spline (degree *k* = 3) provides the necessary flexibility to model the smooth, rounded shape of the spiral while minimizing overfitting or unnecessary oscillations.

The smoothing factor *s* is dynamically adjusted based on the number of data points in the unwrapped spiral, considering the reduced dataset size after the thinning process. The formula used is as follows:

(3) s=(total number of points in unwrapped spiral)×N

where *N* is a scaling factor that controls the degree of smoothing. This adjustment is necessary to maintain a balance between capturing the essential data features and preventing the loss of significant details due to excessive smoothing. Different values of *N* were tested to identify the optimal balance that highlights key data characteristics without obscuring the underlying tremor patterns. The selected value for *N* ultimately influences the smoothness of the LOBF and its ability to accurately represent the tremor deviations.

Table 3 summarizes the impact of various *N* values on the smoothing process, illustrating how fine-tuning this parameter helps to distinguish between normal and Parkinsonian tremor characteristics effectively.

**Figure 6:**
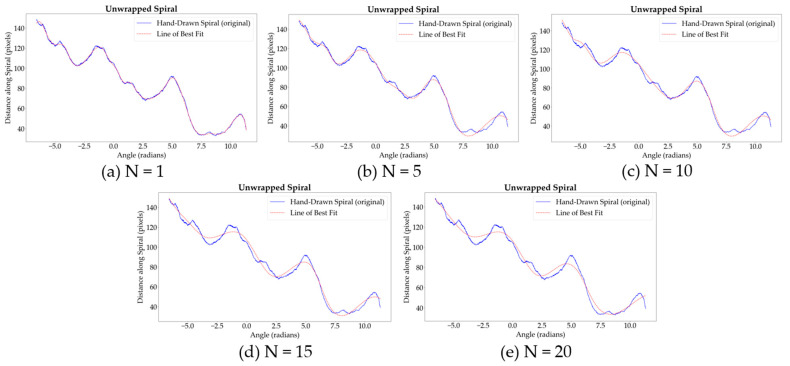
Impact of smoothing degree determined by scaling factor *N.*

Figure 6 illustrates the impact of varying the smoothing parameter s, which is controlled by the scaling factor *N*, on the unwrapped spiral curve. With lower values of *N* (and therefore *s*), the fitted spline closely follows the original spiral, capturing all fine details and minor fluctuations. As *N* increases, the smoothing effect becomes more pronounced, allowing the spline to highlight the significant features of the spiral while reducing sensitivity to less relevant variations, such as noise or minor fluctuations.

The plots generated for each tested value of *N* reveal that lower values (e.g., *N* = 1 and *N* = 5) capture every minor fluctuation, including noise, which may not be meaningful for analyzing tremor characteristics. However, as *N* increases to 10, an optimal balance is achieved: the spline effectively smooths out noise and minor variations while preserving the key features associated with tremor. Further increases in *N* (e.g., *N* = 15 and *N* = 20) lead to excessive smoothing, where the spline risks omitting clinically significant details, as it no longer follows the path of the original spiral closely enough.

Thus, *N* = 10 is identified as the optimal value, providing a balanced approach that allows the spline to accurately reflect the original spiral’s trajectory while applying enough smoothing to distinguish between significant tremor deviations and noise. The resulting curve captures the essential tremor characteristics without over-smoothing, ensuring both fidelity to the original data and clarity in the diagnostic analysis. The tremor deviation is quantified as the difference between the original spiral distances and the distances adjusted by the cubic spline interpolation, as shown in Figure 3 (block 3a). This deviation, plotted as amplitude against angle, reflects variations in hand movement indicative of tremor, with the *x*-axis representing the angle of each point in radians and the *y*-axis indicating the amplitude of deviation.

#### 2.3.5. Frequency Spectrum Analysis

In this study, the FFT is applied to convert the tremor deviation signal from the time domain into the frequency domain. This transformation decomposes the tremor deviation signal into sinusoidal components of different frequencies, allowing for the identification and characterization of dominant frequencies associated with Parkinsonian tremor.

To perform this analysis, the extracted signal is analyzed in the frequency domain using a varying frequency computed for each image as the number of points that form the spiral, divided by the average time it takes to draw a spiral for a healthy person, approximated at 10 s. The FFT is applied on the tremor deviation signal. The magnitude of the frequency spectrum is determined by computing the modulus of the complex values obtained from the FFT. To ensure the accuracy of the analysis across signals of varying lengths, the magnitude of the spectrum is normalized by dividing it by the length of the signal. This normalization step allows for a consistent interpretation of amplitude regardless of the signal length. Without normalization, differences in signal length could lead to discrepancies in the magnitudes, negatively impacting the ability to identify frequency characteristics associated with Parkinsonian tremor.

The FFT produces a set of frequency components, each corresponding to a specific frequency. The frequency vector contains the actual frequency values for each component in the FFT output, allowing each component to be associated with its respective frequency. As the FFT spectrum is symmetric, with the negative half mirroring the positive half for real-valued signals, only the positive half of the spectrum is retained in order to avoid redundancy. This is achieved by keeping the first half of both the frequency and magnitude vectors, ensuring that the resulting spectrum contains only the relevant frequencies for further signal analysis.

#### 2.3.6. Frequency Feature Calculation

Using the data from the frequency domain, several key features were calculated to analyze the tremor signal associated with PD.

- Peak Magnitude: Peak magnitude represents the highest value of the magnitude spectrum, indicating the maximum amplitude of the frequency components in the signal. This peak is determined as the maximum value in the positive magnitude spectrum.
- Peak Frequency: The peak frequency is the frequency at which the magnitude peak occurs, providing insight into the dominant frequency characteristic of the analyzed signal. It is identified by the index of this peak within the frequency vector.
- SNR: The SNR assesses the quality of the signal relative to the noise level. It is defined as the ratio of the average power of the signal to the average power of the noise. The average power of the signal is computed as the sum of the squares of the values above a noise threshold, divided by the number of signal samples, and similarly for the noise. The noise threshold is set as twice the mean of all values in the positive frequency spectrum, rounded to three decimal places. Components of the magnitude vector that exceed this threshold are considered part of the useful signal, while those below are classified as noise, allowing for a clear separation between signal and background noise.

(4) $$SNR=\sum \frac{{\left(signal\;values\right)}^{2}}{len(signal)}/\sum \frac{{\left(noise\;values\right)}^{2}}{len(noise)}-1$$

- Variance: The variance of the signal content was calculated for the segment of the frequency spectrum identified above the noise threshold. This calculation provides an indication of the uniformity and stability of the signal by evaluating how much the magnitude of the frequency components deviates from their mean.
- Bandwidth: The bandwidth represents the total number of sample points contained within the signal, specifically the points above the noise threshold. This measure indicates the range of frequencies that significantly contribute to the signal.

**Figure 7:**
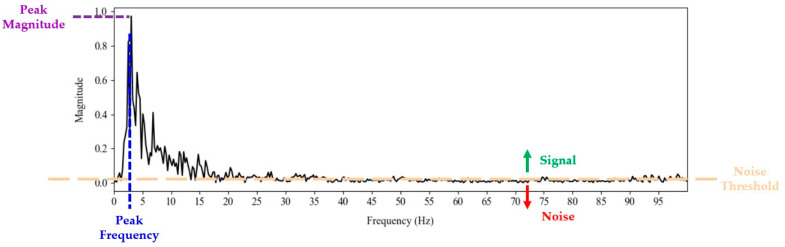
Visualization of calculated frequency features: peak frequency is the frequency at which the peak magnitude is achieved.

These characteristics are illustrated in Figure 7. The peak magnitude represents the highest value of the magnitude spectrum, while the peak frequency is the frequency at which this peak occurs. The *variance*, *SNR*, and *bandwidth* are calculated based on the content of the signal and noise. The *noise threshold* is depicted in orange, separating the significant frequency components (signal) from the background noise. Signal values are above the noise threshold as indicated by the green arrow, indicating the portions of the spectrum considered as signal, while noise values are below the noise threshold, as indicated by the red arrow.

### 2.4. Feature Extraction: Pencil Features

Due to the morphological thinning process necessary to obtain the unwrapped spirals, all detailed information related to the actual pencil content is lost, including the thickness of the drawn line and the pressure applied with the pencil. These features may be significant, as they provide insight into the drawing behavior of patients with PD (e.g., greater pressure results in darker spirals, while lighter pressure results in lighter spirals). To capture these details, in addition to frequency features, pencil-specific features were extracted.

The pencil features were derived from the grayscale images using a technique based on pixel intensity thresholding. Since the majority of each image consists of lighter areas (i.e., the white paper on which the spirals were drawn), the initial threshold value was determined by calculating the mean intensity of all pixels. This initial threshold was further refined to account for potential noise or artifacts from the scanning process, such as shading or minor impurities. To achieve this, an error margin was computed by considering the difference between the initial threshold and the maximum possible pixel intensity value (255, representing white). This error margin was subtracted from the initial mean intensity to obtain a final, adjusted threshold.

Using this refined threshold, a Boolean mask was created to select all pixels in the image with an intensity lower than the final threshold, effectively isolating the pencil-drawn areas from the background. The remaining pixels, representing the actual pencil content, were then used to compute the desired features:

- Pencil thickness: This was calculated as the total number of pixels that constitute the pencil strokes in the image divided by the length of the spiral. The length of the spiral was computed using the sum of the arc lengths of all detected contours in the image.
- Pencil pressure: This was determined by calculating the average intensity of the pixels corresponding to the pencil strokes, providing a measure of the pressure applied during drawing.

**Figure 8:**
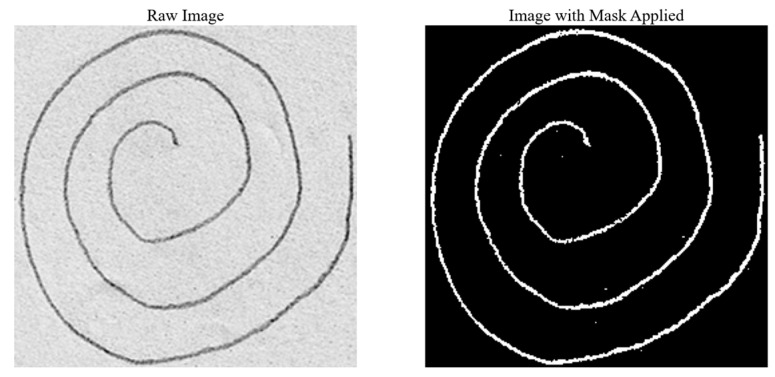
Process of highlighting pencil features.

This process is illustrated in Figure 8.

### 2.5. Classification Models

Using the features extracted as presented in Section 2.3 and Section 2.4, ML techniques for binary classification were performed to differentiate between the drawings produced by healthy subjects and those by PD patients.

The final step for both feature extraction pipelines was storing the features in csv files. Each csv file contained the label for each image and the corresponding values obtained for each feature. These csv files, created for each type of feature, were subsequently used as input data for the classification algorithms.

To ensure data integrity, rows containing NaN (Not a Number) values—meaning they could not be computed for the corresponding drawing—in key feature columns are removed from the csv files. This cleaning step is essential to avoid incomplete or erroneous entries that could negatively impact the model’s performance.

The original dataset is already divided into two subsets, labeled as testing and training. The subset labeled as ‘training’ was used for training and testing and the subset labeled as ‘testing’ was used for validation.

The following ML algorithms were employed for this task SVM, RFs, DT, XGBoost, AdaBoost with DTs, and CatBoost. These algorithms were applied to the three sets of features: frequency domain features (noted as F features), pencil-related features (noted as P features), and a combination of both (noted as FP features).

### 2.6. Model Evaluation

The following evaluation metrics were used to assess the quality of the classification results for each model:

Accuracy: *Accuracy* is defined as the proportion of correctly classified instances over the total number of instances. It is computed as follows:

(5) $$Accuracy=\frac{TP+TN}{TP+TN+FP+FN}$$

where *TP* represents the number of true positives, *TN* the number of true negatives, *FP* the number of false positives, and *FN* the number of false negatives.

Precision: *Precision* measures the proportion of positive instances correctly identified by the classifier and is calculated as follows:

(6) $$Precision=\frac{TP}{TP+FP}$$

Recall (Sensitivity): *Recall*, or *sensitivity*, represents the proportion of actual positive instances that were correctly identified by the model and is computed as follows:

(7) $$Recall=\frac{TP}{TP+FN}$$

F_1_-Score: The *F_1_-score* provides a harmonic mean of precision and recall and is particularly useful when dealing with imbalanced classes. It is calculated as follows:

(8) $$F_{1}\text{-}Score=\frac{2TP}{2TP+FP+FN}$$

For the best-performing model, a confusion matrix is also presented to provide a detailed evaluation of its classification performance, offering insight into the distribution of true positives, true negatives, false positives, and false negatives.

## 3. Results

This section presents the performances of the feature extraction approaches presented in the Methods Section. Classification models for differentiating between drawings produced by healthy subjects or PWP subjects are built using only the frequency domain features (noted as F features), only the pencil-related features (noted as P features), and using both types of features (noted as FP features). Multiple classification algorithms are built using the three types of features. Results are presented for each approach in terms of overall accuracy. For the best approach, detailed performances are presented in terms of *accuracy*, *precision*, *recall,* and *F_1_-score*, along with the confusion matrix.

For the six ML algorithms that were employed (the three standard classifiers SVMs, RF, and DT and three boosting methods—XGBoost, AdaBoost with DT, and CatBoost), a grid search with five-fold cross-validation (*cv* = 5) was performed to systematically explore the hyperparameter space. This approach was chosen to ensure the selection of the optimal parameters that yield the best performance for each model. Grid search with cross-validation helps to prevent overfitting and ensures that the chosen hyperparameters lead to the most accurate and robust results. During the five-fold cross-validation, normalization is performed individually for each training fold. For each fold, normalization is performed to the training data of the current fold, ensuring the scaling parameters are derived solely from the training data. The scaler fitted on the training fold is afterwards utilized to normalize the corresponding validation set. This method prevents any leakage of information from the testing set into the training process.

The results obtained for each classification algorithm and feature set are summarized in Table 4.

Across all classifiers, the FP feature set, which combines both frequency and pencil features, consistently outperformed the individual F and P sets in almost every metric. This confirms the hypothesis that combining both frequency- and pencil-related features leads to more accurate models. Specifically, in most cases, the FP set yielded the highest *accuracy*, *precision*, *recall*, and *F_1_-scores* compared to using either feature set in isolation. The better performances of the FP set may be attributed to the complementary nature of the features extracted. Frequency-based features provide insight into the tremor characteristics of the patient’s hand movements, while pencil-based features capture subtleties in line pressure and thickness that may be indicative of bradykinesia or rigidity. By using both types of information, the algorithms take into account the three main motor symptoms associated with PD.

Among the classifiers, RFs demonstrated the highest overall performance, especially when using the FP feature set. With an accuracy of 95.24%, 95.67% precision, and a 95.24% recall, this algorithm achieved the best results across all evaluated models. Its high F1-score of 95.24% further confirms the model’s ability to balance precision and recall, making it the most reliable choice for this task.

**Figure 9:**
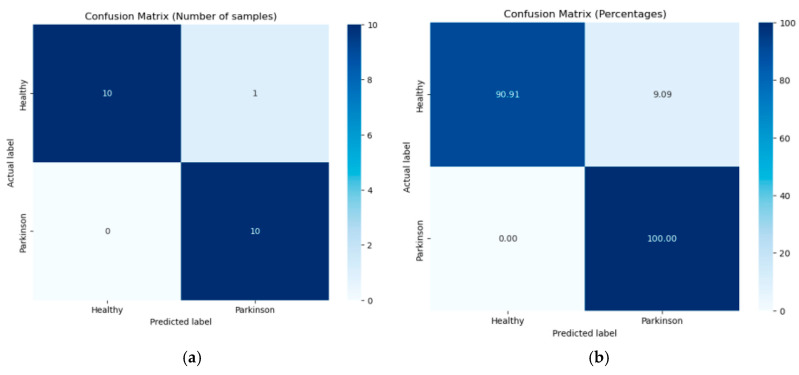
Confusion matrices for the RF classifier using the FP feature sets: (**a**) values shown as number of samples in each category and (**b**) values shown as percentages.

Given the superior performance of RFs, a more detailed analysis of the classification results on the testing data is presented in the form of a confusion matrix. The confusion matrices, shown in Figure 9 in both percentage form and as number of samples, offer more details about the model’s ability to correctly classify PD versus healthy samples and highlights the false positive and false negative rates, providing a basis for further improvements.

The model correctly classified 10 images (90.91%) from healthy subjects (true negatives) and 10 images (100%) from PD patients (true positives). However, one healthy image was incorrectly classified as PD (false positive).

These results demonstrate the model’s strong ability to accurately identify both healthy and PD cases, particularly with a low rate of false negatives, which is essential for minimizing missed diagnoses in the context of PD detection. This analysis provides a solid foundation for the model’s utility in clinical applications and in various testing environments. The high classification accuracy makes this model applicable for practical use in the early detection of PD.

## 4. Discussion

The findings of this study highlight the potential for using graphical analysis of patient drawings as a low-cost, accessible tool for PD detection. By converting spiral drawings into the frequency domain, the work demonstrates the potential of a simple, accessible method to identify motor dysfunctions commonly associated with PD.

The method used in this study provides several advantages. The simplicity of drawing spirals on paper with a regular pencil makes it a low-cost and easily implementable method in both clinical settings with limited access to advanced technology and at home. This contrasts with previous studies that employed digitized tools such as graphic tablets or sensor-equipped pens [6,12,14,15,16,17], which, while highly accurate in some approaches, may not be readily available in all testing environments. From the perspective of the existing literature, previous studies have demonstrated the utility of digitized approaches for analyzing motor symptoms in PD patients, often focusing on precision and fine motor control. As such, the method demonstrated in this study offers a viable alternative for early detection and monitoring of PD, especially in resource-limited settings. The innovative aspect of the feature extraction process is the conversion of the hand-drawn spirals into frequency spectra, which enhances the accuracy of diagnosis without requiring complex data acquisition setups, providing a cost-effective and efficient method. The integration of this model into the NeuroPredict platform better supports its aim by providing an affordable tool for managing neurodegenerative diseases.

The performance achieved by the selected PD detection model exceeds the performances of other works in the field, and it is similar to the state of the art. The highest accuracy reported found in the literature review is 96.02% [14]. The approach presented in the current work achieves an average accuracy of 95.24%, a difference of less than 1%. However, it has the clear advantage of not requiring specialized devices for data acquisition when testing new data. In contrast, the work presented in [14] relies on kinematic features that can only be extracted when the drawings are performed using a digitized graphics tablet.

The best result for methods that do not require specialized devices for acquiring test data, as found in the literature review, reports an average accuracy of 96% [8], close to the 95.24% average accuracy achieved by the method proposed in the current work. Comparing the two works, both approaches have advantages and disadvantages: the proposed method employs RFs while in [8] a DL approach is employed, namely a DenseNet network with an unspecified number of layers. Regarding this aspect, DenseNet is more complex, requiring greater computational power and time for both training and testing new data. On the other hand, DenseNet is directly fed new images, requiring some preprocessing steps, depending on the implementation. The approach presented in this work is based on carefully crafted features that must be computed on new images before being used as inputs to the classification algorithm. As a conclusion to this comparison, RFs are more suited for real-time applications and resource-constrained environments considering that they are faster and more efficient in computing and storage usage.

**Figure 10:**
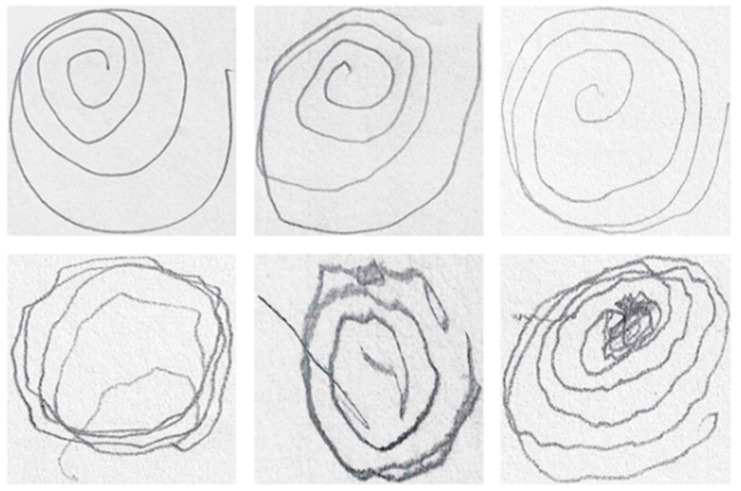
Example spirals that could not be unwrapped by the proposed method.

While the results offer significant insights, several limitations and areas for improvement need to be considered. One of the primary limitations stems from the morphological thinning and unwrapping processes, which restricted the suitability of some images for analysis. Specifically, intersecting or closely grouped lines in some spirals, as well as inconsistencies in the number of rotations drawn by participants, resulted in the exclusion of certain images from the dataset. These constraints reduced the number of valid samples, potentially affecting the generalizability of the findings. Examples of unusable images due to these issues are provided in Figure 10.

A potential solution to this limitation could involve the development and implementation of advanced algorithms capable of detecting intersections and applying automated corrections, such as reconstructing missing or overlapping segments of the spirals. By refining the image processing techniques, future studies could address these issues and include a greater variety of spirals, leading to more comprehensive datasets and improved diagnostic accuracy. In addition, further exploration into the use of other types of graphical representations, such as different geometric shapes or freehand movements, may provide additional diagnostic insights. As the disease affects different aspects of motor control, testing various tasks could help capture a broader spectrum of symptoms.

Another limitation was the relatively small size of the dataset. To overcome this, future research should aim to collect larger and more diverse datasets, including drawings from a broader range of subjects with varying degrees of PD severity and from different clinical environments. Such an approach would not only improve the robustness of the findings but also enhance their applicability across different patient populations. Collaboration with multiple medical institutions and researchers could facilitate access to more extensive data, ultimately supporting the generalization of the proposed method.

Future research should focus on improving the robustness of the image analysis algorithms to handle more complex and imperfect spiral drawings, as well as expanding the dataset to include more diverse and representative patient populations. The final algorithm will be integrated in the NeuroPredict platform for detecting and managing neurodegenerative diseases. The presented algorithm will work alongside multiple diagnostic and monitoring algorithms that rely on multimodal data, enabling a comprehensive assessment of the tested and monitored subjects.

## 5. Conclusions

The presented study successfully demonstrates the potential of using a simple automated test for detecting signs of PD. It demonstrates the potential of AI in improving early detection of PD by analyzing hand-drawn spirals. The algorithmic approach relies on converting the spiral drawings into frequency spectra, extracting both frequency-derived and pencil content features.

The algorithm achieved an overall accuracy of 95.24%, a high accuracy comparable with the state of the art identified in the literature review. One of the key advantages of the proposed method is that it does not require specialized devices like graphic tablets or digital pens. The test can be conducted using only a pencil and paper, making it accessible in diverse environments, including resource-limited settings. This makes it feasible for broader use outside clinical settings, including in home environments.

The presented algorithm is designed to be integrated into the NeuroPredict platform, a complex medical platform that aims to integrate predictive models based on AI with data from IoMT devices and self-reported data to manage aspects related to three neurodegenerative diseases, including PD.

The innovative aspect of this study lies in the feature extraction pipeline, where frequency features are extracted directly from the drawn images, without additional information recorded using specialized devices.

Some limitations are noted regarding the relatively small dataset and the fact that the feature extraction algorithm is not appropriate for some of the spiral images presented. Future research will aim to increase the dataset size and improve the algorithm so that it can handle all types of spirals.

The presented approach contributes to the field by showing that valuable insights can be obtained using non-digital drawing methods. This opens up new possibilities for scaling PD detection methods in settings where digital infrastructure is lacking. Furthermore, this method could make screening for PD more accessible, allowing for an initial assessment without the need for it to be performed by highly trained personnel.

## Figures and Tables

**Table 1:** Comparative presentation of other works on PD detection from hand-drawn Archimedean spirals.

Previous Work/Year of Study	Dataset Used	Features	Classification Algorithm Basis	Best Average Accuracy [%]	Test Data May Be Acquired with Pencil and Paper
Proposed method/ 2024	Zham et al. [7]	Frequency-based features combined with pencil content features	RFs	95.24	Yes
[10] 2024	Zham et al.	DL directly on images from the augmented dataset	DenseNet201	94	Yes
[8] 2024	Zham et al.	DL directly on images from the augmented dataset	DenseNet—50 epochs	96	Yes
			DenseNet—100 epochs	89	
[6] 2022	University of California, Irvine Parkinson’s disease spiral drawings [11]	Kinematic features	k-Nearest Neighbors	92.38	No
			RFs	92.32	
[12] 2023	Built by the authors for the study: 58 images	Kinematics features Pressure (force) features	CatBoost	94.83	No
			LightGBM	89.65	
			RFs	79.31	
			Logistic regression	84.48	
[13] 2020	Zham et al.	DL directly on images from the augmented dataset	Convolutional Neural Networks	93.33	Yes
[14] 2021	University of California, Irvine Parkinson’s disease spiral drawings	Kinematic features	RFs	94.35	No
			Support Vector Machine (SVM)	93.58	
			AdaBoost	96.02	
			Extreme Gradient Boosting	89.80	
[15] 2019	PaHaW	Tremor estimation distance feature	SVM	75.92	No
		Features based on Fourier Transform	SVM	79.63	
		Both above categories	SVM	81.66	
[16] 2018	PaHaW	Kinematic, pressure and spatio-temporal features	Gaussian Naïve Bayes	54.67	No

**Table 2:** Steps for obtaining the frequency domain features.

Step	Description	Reference Block in Figure 3
1	Load the original image of the drawn spiral	Block 1
2	Perform morphological thinning to obtain one-pixel-width spirals	Block 1a
3	Unwrap the spiral and obtain the “Line-of-Best-Fit (LOBF)”	Block 2
4	Compute the tremor deviation by taking the difference between the LOBF and the spiral drawing	Block 3a
5	Convert the tremor deviation signal to the Fourier domain	Block 3b
6	Compute the relevant frequency features: peak magnitude, peak frequency, variance, SNR, and bandwidth	Block 4

**Table 3:** Values tested for the scaling factor N to determine the impact on the smoothing process.

Parameter	Tested Values
Scaling factor (*N*)	1, 5, 10, 15, 20

**Table 4:** Classification results using only the frequency-related features (noted as F), only the pencil-related features (P), and the combined feature set (noted as FP).

Algorithm	Feature Set	Accuracy [%]	Precision [%]	Recall [%]	F_1_-Score [%]
DT	F	57.14	57.40	57.14	57.14
	P	56.57	76.79	56.67	46.65
	FP	90.48	90.48	90.48	90.48
RF	F	90.48	90.48	90.48	90.48
	P	70.00	70.09	70.00	69.97
	**FP**	**95.24**	95.67	95.24	95.24
SVM	F	80.95	81.96	80.95	80.69
	P	66.67	67.94	66.67	66.06
	FP	85.71	85.98	85.71	85.65
XGBoost	F	76.19	78.23	76.19	75.52
	P	50.00	25.00	50.00	33.33
	FP	85.71	85.98	85.71	85.65
AdaBoost with DT	F	90.48	90.48	90.48	90.48
	P	70.00	70.00	70.00	69.97
	FP	90.48	90.48	90.48	90.48
CatBoost	F	80.95	80.95	80.95	80.95
	P	56.67	56.94	56.67	56.23
	FP	80.95	80.95	80.95	80.95

## Data Availability

The dataset used in the current work has been made available by the original authors and is referenced in the References Section at position [7].
